# Several Nuclear Events during Apoptosis Depend on Caspase-3 Activation but Do Not Constitute a Common Pathway

**DOI:** 10.1371/journal.pone.0006234

**Published:** 2009-07-29

**Authors:** Lisa Trisciuoglio, Marco Emilio Bianchi

**Affiliations:** 1 Chromatin Dynamics Unit, San Raffaele Scientific Institute, Milano, Italy; 2 Università Vita Salute San Raffaele, Milano, Italy; Universidade de Sao Paulo, Brazil

## Abstract

A number of nuclear events occur during apoptosis, including DNA laddering, nuclear lamina breakdown, phosphorylation of histones H2B and histone H2AX, and the tight binding to chromatin of HMGB1 and CAD, the nuclease responsible for DNA laddering. We have performed an epistasis analysis to investigate whether these events cluster together in pathways. We find that all depend directly or indirectly on caspase-3 activation. CAD activation, H2AX phosphorylation and DNA laddering cluster together into a pathway, but all other events appear to be independent of each other downstream of caspase-3, and likely evolved subject to different functional pressures.

## Introduction

Apoptosis entails a complex set of events, which must have evolved to provide a specific advantage to the individual. However, while the ultimate advantage of cell apoptosis (favouring in some way the other cells of the organism of the population) is clear, less clear is the advantage provided by each of the events occurring during apoptosis, including chromatin condensation, DNA laddering and nuclear fragmentation.

Fragmentation of DNA to nucleosome-sized fragments, or DNA laddering, is executed by activated DNA nuclease (CAD) after caspase-dependent cleavage of the CAD inhibitor, ICAD [1]. *Cad−/−* mice have reduced DNA fragmentation during cells apoptosis, but show no gross developmental abnormality and are normally fertile [2]. Besides CAD, Endonuclease G released from the mitochondria and more than 20 other enzymatic activities have been implicated in apoptotic DNA cleavage [3].

Chromatin condensation, or pyknosis, is morphologically evident, but does not cause the “collapse” of chromatin. Condensation by itself does not impair protein mobility: GFP and most nuclear proteins, including HMGN1, HMGN2, NF1, and histone H1 retain their mobility in condensed chromatin [4]. Interestingly, only two proteins appear to be immobilized in condensed apoptotic nuclei: CAD [5] and High Mobility Group Box 1 (HMGB1) [4]. HMGB1 is an abundant protein with several roles in transcription, recombination and chromatin organization [6]. Cells dying in a traumatic way release HMGB1 in the extracellular space, where it signals tissue damage, trigger inflammation, activate innate and adaptive immunity responses, and promote tissue repair (reviewed in [7]). We have thus proposed that apoptotic cells actively modify their chromatin to retain HMGB1 and avoid issuing an inflammatory signal [8].

Nuclear fragmentation, or karyorrhexis, occurs because of lamin A cleavage by caspase-6 [9]. Mice lacking lamin A/C develop to term with no overt abnormalities, although their postnatal growth is severely retarded and is characterized by the appearance of muscular dystrophy [10].

Several post-translational modifications of histones also occur during apoptosis. Phosphorylation of histone H2B appears to be linked to nuclear condensation, both in yeast and mammalian cells [11], [12]. Phosphorylation of histone H2AX at Tyr142 and Ser139 was recently shown to be essential to decide whether to repair a DNA damage or to promote apoptosis [13]. Histones H3 and H4 are deacetylated during apoptosis, and inhibition of histone deacetylases with trichostatin A interferes with HMGB1 immobilization [4].

Given the variety of nuclear events occurring in apoptosis, we have used a typical epistasis analysis, investigating whether blocking any of the events affects any of the others. In particular, we were interested to determine possible interconnections between histone modifications and morphological changes. In the most extreme outcome, all events might belong to a single cellular pathway, and then would have evolved together to provide a single advantage. We find the opposite: most events occur independently of each other, and thus are separate endpoints, direct or indirect, that follow the activation executioner caspases.

## Results

### HMGB1 immobilization in apoptotic nuclei

**Figure 1:**
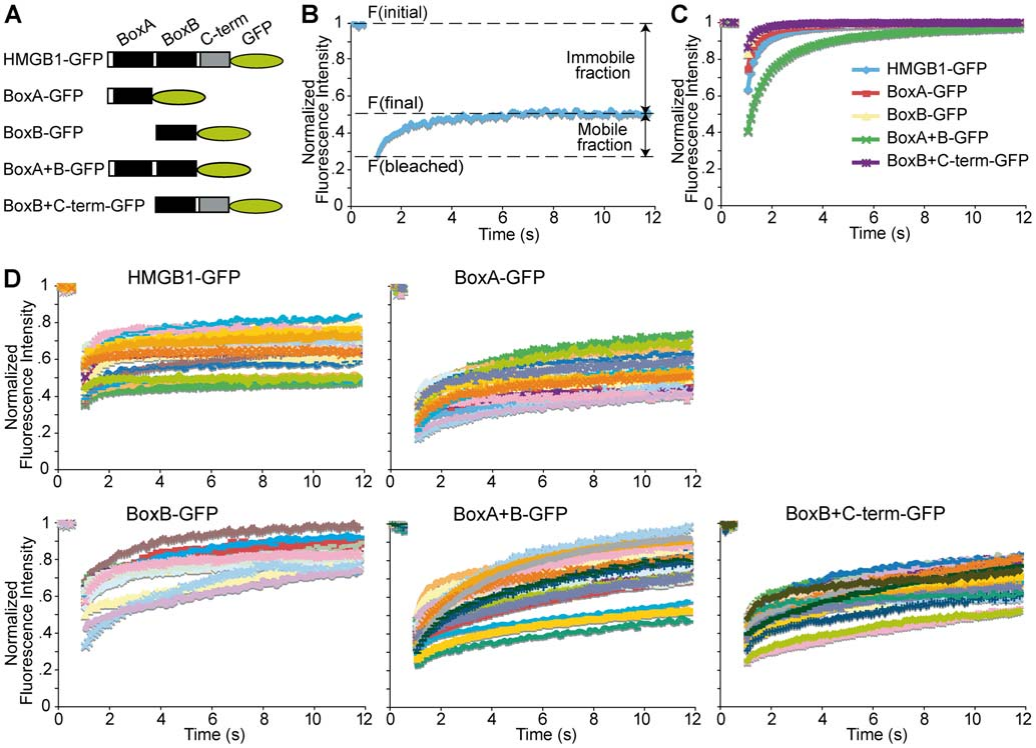
The entire HMGB1 protein is required for its immobilization onto apoptotic chromatin. (A) Schematic representation of the GFP fusion proteins. (B) A typical FRAP from one cell. Fluorescence intensity in the bleached spot is plotted as a function of time. The typical parameters of a FRAP experiment are shown. (C) FRAP experiments performed in proliferating HeLa cells transiently expressing the various GFP fusion proteins. Each line corresponds to the average of fluorescence recovery in at least 20 cells. The standard deviation is about 5% over most data points, and is not shown to avoid clutter. (D) FRAP experiments performed in apoptotic HeLa cells transiently expressing the various GFP fusion proteins. Each line corresponds to the fluorescence recovery measured in a different cell. Individual cell traces are shown, rather than their average, because of the high variation among apoptotic cells.

The immobilization of HMGB1 and CAD during apoptosis has been demonstrated using GFP fusion proteins and laser photobleaching [4], [5]. FRAP (fluorescence recovery after photobleaching) entails photobleaching of a small spot in the nucleus, followed by repeated imaging of the photobleached spot to measure the recovery of fluorescence within it (and thus the mixing of non-photobleached and photobleached but fully functional molecules from adjacent areas in the nucleus). FRAP is a time-lapse experiment: the fluorescence in the bleach region is monitored during a pre-bleach period to determine the initial fluorescence intensity F(initial), immediately after the bleaching F(bleached) and until it reaches a final value F(final), when no further increase can be detected (Figure 1B).

We confirmed, using FRAP, that in living HeLa cells the recovery of HMGB1-GFP fluorescence is complete and fast; 80% recovery is obtained in about 1.5 seconds (Figure 1C). In cells treated with TNF-α and cycloheximide (CHX) in order to induce apoptosis, the recovery of fluorescence of HMGB1-GFP is only partial and always reaches a plateau (Figure 1D). The amount of immobilized HMGB1-GFP is indicated by the difference between the plateau level and the 100% recovery limit, and varies among different apoptotic cells, possibly depending on the heterogeneity in the progression of the apoptotic process in the cells being imaged.

We then evaluated the mobility of progressive COOH-terminal truncations of HMGB1, fused in frame to EGFP (Figure 1A). Western blot analysis verified the expected molecular mass of the truncated HMGB1-GFP fusion proteins (data not shown). In living cells, BoxA+B-GFP recovers 80% of initial fluorescence after about 2.5 seconds, while BoxA, BoxB and BoxB+C-term truncated proteins were slightly faster than the full-length protein, in agreement with the notion that the two HMG boxes cooperate in binding to chromatin, and that the acidic tail reduces the DNA binding activity of the HMG boxes.

The mobility of the various deletion mutants was reduced in apoptotic cells as compared to living cells (Figure 1C), as expected. However, their immobilization in apoptotic cells was not complete: fluorescence recovery did not reach a plateau, at least within 12 seconds (Figure 1D). Thus, all fragments bind rather tightly to apoptotic chromatin, but not as tightly as full-length HMGB1.

**Figure 2:**
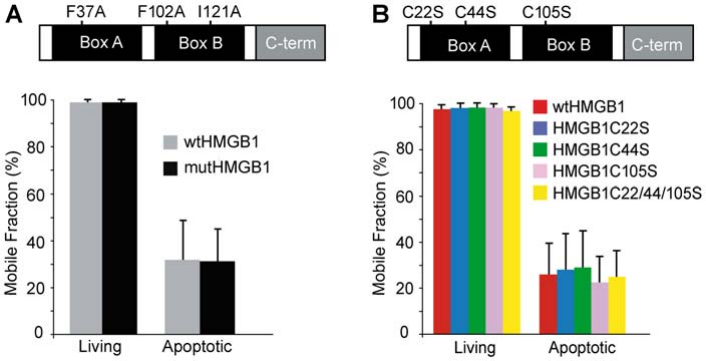
HMGB1 immobilization is not mediated by binding to DNA or disulfide bonds. (A) The substitution of F37, F102 and I121 residues with alanines do not impact HMGB1 apoptotic immobilization. FRAP experiments were performed in proliferating and apoptotic HeLa cells transfected respectively with wt and mutHMGB1. The results are expressed as mean +/− standard deviation (n = 16). (B) The three cysteine residues in HMGB1 were mutated to serine. FRAP experiments were performed in proliferating and apoptotic HeLa cells transfected respectively with wtHMGB1-GFP, singly mutated HMGB1 (HMGB1C22S-GFP, HMGB1C44S-GFP and HMGB1C105S-GFP), and triply mutated HMGB1 (HMGB1C22/44/105S-GFP). The results are expressed as the mean +/− standard deviation (n = 17).

The two HMG boxes have hydrophobic residues in conserved positions (F37, F102, I121) that anchor them to the minor groove of DNA [14], and three cysteine residues, of which two (Cys22 and Cys44) can form a disulfide bond, while the third (Cys105) is free (Figure 2) [15]. Triple-mutated HMGB1-GFP (mutHMGB1-GFP), where the 3 critical hydrophobic residues have been replaced by alanine, was already shown to have reduced binding to chromatin [16], [17]. We also constructed cysteine-to-serine single and triple mutants. We tested each mutant by FRAP in proliferating HeLa cells and in cells forced to undergo apoptosis. For all HMGB1 mutants the mobile fraction in apoptotic cells corresponds roughly to 25% of the total pool of fluorescent protein, not unlike the wt (Figure 2). Therefore, the severe reduction of either the ability to bind DNA or to form disulfide bonds does not impair HMGB1 immobilization in the nucleus of apoptotic cells. These data also imply that oxidation of HMGB1, that occurs on C105 [18], does not modify HMGB1 binding to chromatin.

### Caspase-3 activation determines HMGB1 immobilization and histone H2B phosphorylation

Activation of the executioner caspase-3 is a key event in apoptosis. Activated caspase-3 cleaves ICAD and releases active CAD, which cleaves the DNA to nucleosome-sized fragments [1]. CAD is then immobilized in apoptotic nuclei [5]. Moreover, activated caspase-3 cleaves inactive Mst1 and converts it to an enzymatically active form that is transported to the nucleus and phosphorylates histone H2B on serine 14 [12]. We then investigated whether caspase-3 activation also controls HMGB1 immobilization and H2AX phosphoryation.

**Figure 3:**
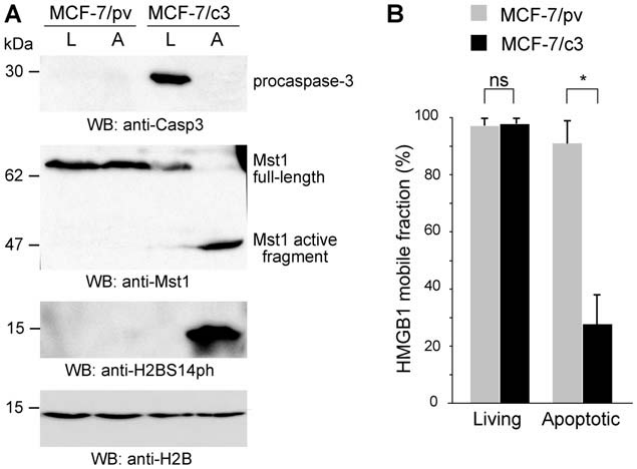
The lack of caspase-3 expression in MCF-7 cells abolishes H2BS14 phosphorylation and preserves HMGB1 mobility in the nucleus of apoptotic cells. (A) Proliferating (L) and apoptotic (A) MCF-7/pv and MCF-7/c3 cells were probed by Western blotting. MCF-7 cells, which lack caspase-3, do not cleave Mst1 during apoptosis and do not phosphorylate histone H2B. Mst1 cleavage and H2B phosphorylation are re-established after caspse 3 re-expression. (B) FRAP experiments were performed in proliferating and apoptotic MCF-7/pv and MCF-7/c3 cells transiently transfected with HMGB1-GFP. The results are expressed as the mean +/− standard deviation (n = 16). The asterisk indicates that the difference in the fraction of mobile HMGB1-GFP between apoptotic MCF-7/pv and MCF-7/c3 cells is highly significant (P<0.001, unpaired t-test).

In 3T3 fibroblasts, the caspase inhibitor z-DEVD-fmk abrogated HMGB1 immobilization in dying cells (data not shown). We then used MCF-7 cells, which lack functional caspase-3 [19], reconstituted with a plasmid expressing caspase-3 (MCF-7/c3) or with the pBabe/puro empty vector (MCF-7/pv) as a control [20]. We first showed that Mst1 cleavage and H2B S14 phosphorylation occurred only in MCF-7/c3 cells and not in MCF-7/pv cells (Figure 3A). However, both MCF-7/c3 and MCF-7/pv cells died after stimulation with TNF-α and CHX, either by apoptosis (MCF-7/c3 cells) or a different caspase-3–independent mode of death that produces extensive morphological changes including swelling and detachment from the solid support (MCF-7/pv cells) (data not shown). We then investigated the mobility of HMGB1 in dying cells treated with TNF-α and CHX: only 20% of HMGB1-GFP remains mobile in MCF-7/c3 cells, while essentially all HMGB1 remains mobile in MCF-7/pv cells (Figure 3B).

Taken together, these results indicate that in MCF-7 cells both H2B S14 phosphorylation and HMGB1 immobilization to apoptotic chromatin are caspase-3–dependent events.

### H2AX S139 phosphorylation is a caspase-3 dependent event in TNF-α induced apoptosis, but does not mediate HMGB1 immobilization

Recently Lu et al. [21] demonstrated that during UVA-induced apoptosis H2AX is phosphorylated by JNK1, and that this post-translational modification is responsible for the induction of apoptosis and for CAD-mediated DNA fragmentation. In contrast to previous results [22], they also demonstrated that UVA-induced H2AX S139 phosphorylation by JNK1 is completely independent from caspase-3 activation.

**Figure 4:**
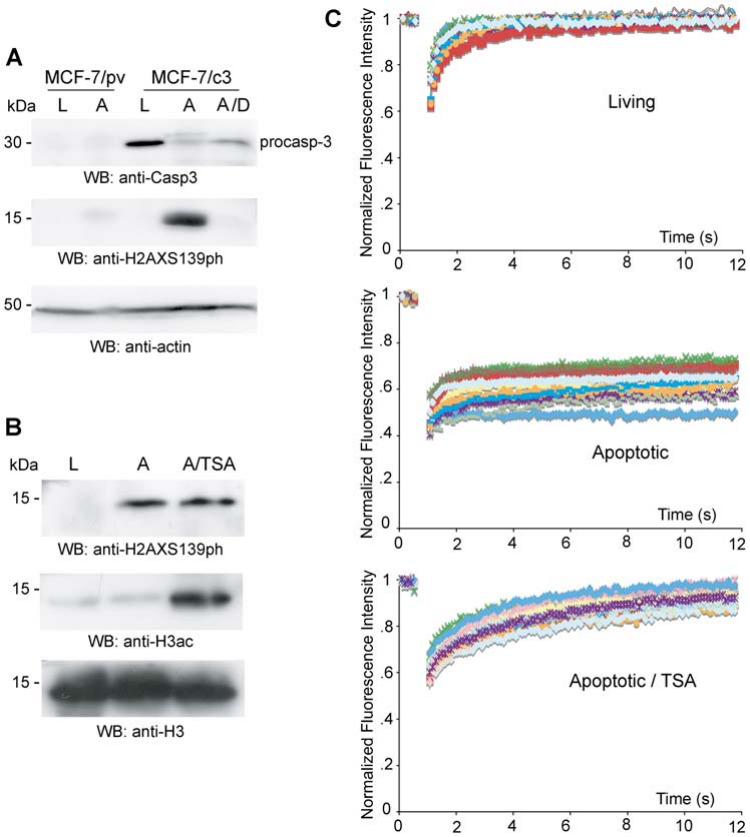
In TNF-α induced apoptosis, H2AX S139 phosphorylation is a caspase-3 dependent event but does not influence HMGB1 immobilization. (A) Western Blot of MCF-7 cells stably transfected with empty vector (MCF-7/pv) MCF-7 cells transfected with caspase-3 (MCF-7/c3). Cell were either proliferating (L) or apoptotic (A), pretreated with z-DEVD-fmk (A/z-D) or not. The absence of caspase-3 or its inhibition abolish H2AX phosphorylation. (B) Western Blot of proliferating (L) and apoptotic (A) HeLa cells, treated with 100 ng/ml TSA just prior to apoptosis (A/TSA) or not. In TSA treated cells H2AX is phosphorylated, but HMGB1 is mobile; thus, the two events do not correlate. (C) FRAP experiments performed in HeLa cells (living, apoptotic, and apoptotic treated with TSA) transiently expressing HMGB1-GFP. Each line corresponds to the fluorescence recovery measured in a different cell.

Given these contrasting data, we investigated whether in TNF-α induced apoptosis H2AX phosphorylation is dependent on caspase-3 activation. We used proliferating and apoptotic MCF-7/pv and MCF-7/c3 cells, pre-treated or not with the caspase-3 inhibitor z-DEVD-fmk. In apoptotic MCF-7/pv cells there is no detectable level of H2AX S139 phosphorylation, while in apoptotic MCF-7 cells reconstituted with caspase-3 (MCF-7/c3) there is a strong induction of this histone modification. Moreover, H2AX S139 phosphorylation is completely abolished by the treatment with z-DEVD-fmk prior to apoptosis induction (Figure 4A). Thus, in TNF-α induced apoptosis H2AX S139 phosphorylation is a caspase-3 dependent event.

Our lab has demonstrated that the treatment of apoptotic cells with the HDAC inhibitor Trichostatin A (TSA) strongly inhibits HMGB1 immobilization to apoptotic chromatin [4]. Thus, we tested the concordance of H2AX S139 phosphorylation and HMGB1 immobilization in TSA-treated apoptotic cells. We found that the treatment of apoptotic cells with TSA induces histone hyperacetylation, but does not influence the level of H2AX S139 phosphorylation. HMGB1 mobility is only modestly slowed down, but not eliminated, in apoptotic cells treated with TSA (Figure 4C).

These results indicate that H2AX S139 phosphorylation alone is not sufficient to immobilize HMGB1.

### Inhibition of Mst1 expression completely abolishes H2B S14 phosphorylation and does not affect DNA laddering or HMGB1 and CAD immobilization in apoptotic HeLa cells

**Figure 5:**
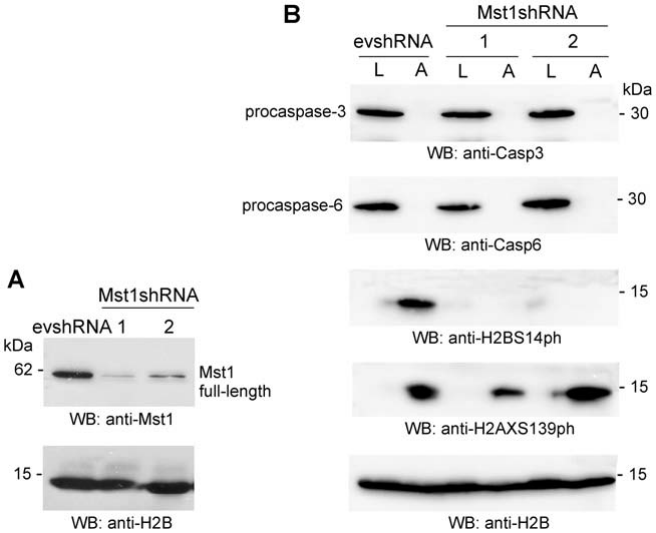
The knockdown of Mst1 abolishes H2B S14 phosphorylation but does not influence either HMGB1 or CAD immobilization in apoptotic HeLa cells. (A) HeLa cells were stably transfected with Mst1shRNA (independent clones 1 and 2) or with the empty vector (evshRNA). Western blotting shows that Mst1 expression is severely reduced in Mst1shRNA HeLa cells. (B) Western blot of living (L) and apoptotic (A) evshRNA and Mst1shRNA cells (clones 1 and 2). Caspase-3 and caspase-6 are cleaved similarly in both cell lines undergoing apoptosis, while H2BS14 phosphorylation is markedly induced only in evshRNA cells and not in apoptotic HeLa cells depleted of Mst1.

To directly test whether H2B S14 phosphorylation could be involved in HMGB1 and CAD immobilization during apoptosis, we abolished by shRNA the expression of the major kinase responsible for H2B phosphorylation, Mst1 (Figure 5A). H2B phosphorylation is markedly induced only in control HeLa cells (stably transfected with empty vector evshRNA) and not in HeLa cells depleted of Mst1, even if both cell lines equally undergo apoptosis, as demonstrated by procaspase-3 and 6 cleavage (Figure 5B). Based on this, we tested the mobility of HMGB1 and CAD in proliferating and apoptotic HeLa Mst1shRNA and evshRNA stable clones. The depletion of Mst1 in HeLa cells undergoing apoptosis does not affect DNA laddering, and does not influence the mobility of CAD and HMGB1, either (Figure S1).

These results indicate that the immobilization of HMGB1 and CAD is not caused by the phosphorylation of H2B at Ser14; likewise, phosphorylation of H2B at Ser14 is not required for DNA laddering.

### HMGB1 depletion does not affect DNA laddering, H2AX and H2B phosphorylation, or CAD immobilization

**Figure 6:**
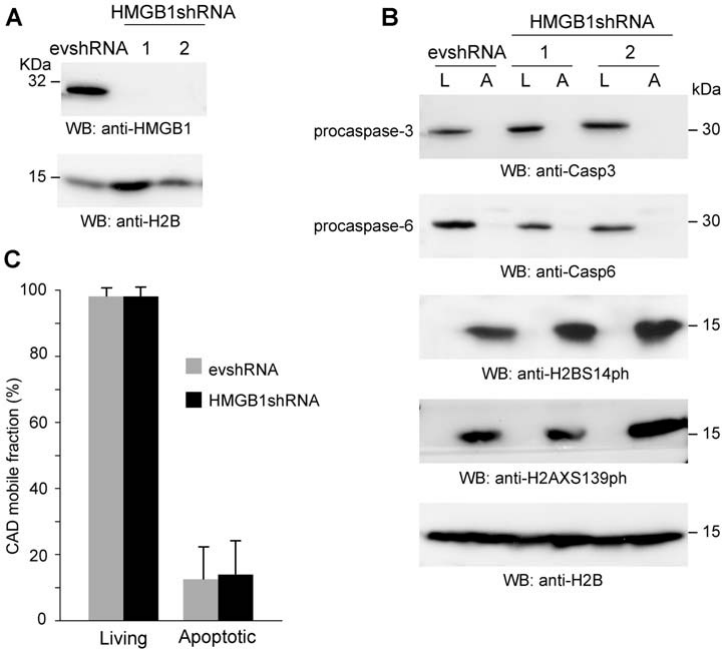
CAD immobilization during apoptosis is not affected in HMGB1shRNA cells. (A) HeLa cells were stably transfected with HMGB1shRNA or with the empty vector (evshRNA) as control. Western blotting shows that HMGB1 expression is abolished in HMGB1shRNA cells. (B) Western blots of living (L) and apoptotic (A) evshRNA and HMGB1shRNA cells show that caspase-3 and caspase-6 are cleaved in both cell lines; H2BS14 and H2AX phosphorylation are clearly induced in both cell lines. (C) FRAP experiments to measure CAD mobility were performed in living and apoptotic evshRNA and HMGB1shRNA cells transfected with CAD-GFP. The results are expressed as the mean +/− standard deviation (n = 20).

Both CAD and HMGB1 are immobilized in apoptotic cells, and might influence each other. To test directly this hypothesis, we generated HeLa cells stably transfected with a vector encoding HMGB1 shRNA (HMGB1shRNA), and with the empty vector as a control (evshRNA). We confirmed by Western Blotting that HMGB1 expression is abolished in cells stably expressing HMGB1 shRNA (Figure 6A). However, HMGB1 depletion does not impair the induction of apoptosis, as demonstrated by procaspase-cleavage and histone post-translational modifications (Figure 6B), or DNA laddering (Figure S1).

We then analyzed CAD-GFP mobility by FRAP experiments in control and HMGB1-depleted HeLa cells. Even in the absence of HMGB1 expression, during apoptosis just a small fraction of the entire pool of CAD-GFP remains mobile (Figure 6C). Thus, CAD immobilization does not depend on the presence of HMGB1.

### HMGB1 and CAD immobilization to apoptotic chromatin do not depend on caspase-6 expression

**Figure 7:**
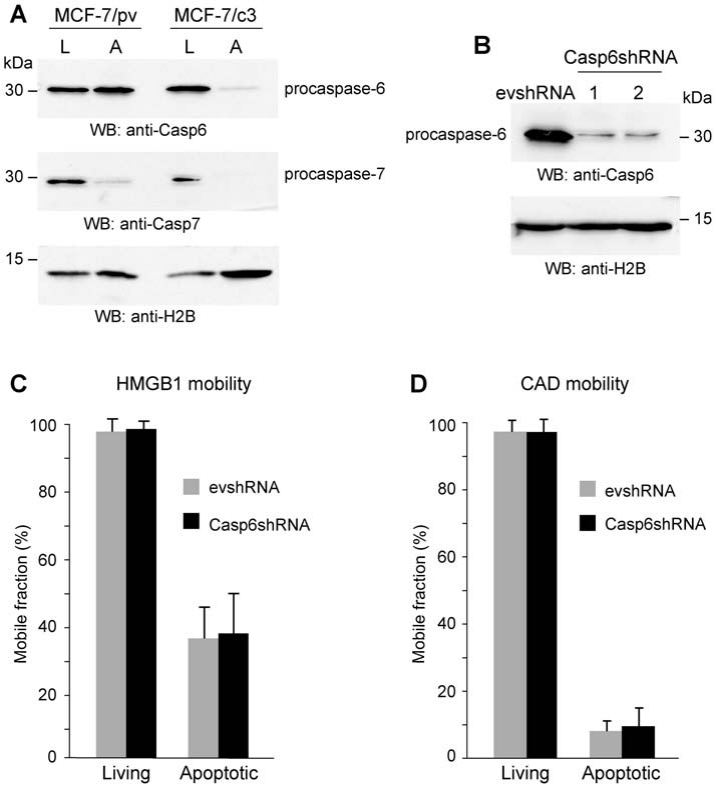
Neither HMGB1 nor CAD immobilization onto apoptotic chromatin depend on caspase-6 expression. (A) Western blot of living (L) and apoptotic (A) MCF-7/pv and MCF-7/c3 cells. Caspase-7, but not caspase-6, is activated in MCF-7/pv forced to undergo apoptosis using TNF-α and CHX. Both caspases are activated in apoptotic MCF-7/c3 cells. (B) HeLa cells were stably transfected with Casp6shRNA or with the empty vector (evshRNA). Western blots show that caspase-6 expression is severely reduced in casp6shRNA HeLa cells. (C) FRAP experiments were performed in living and apoptotic evshRNA and casp6shRNA HeLa cells transiently transfected with HMGB1-GFP. The results are expressed as the mean +/− standard deviation (n = 20). (D) FRAP experiments were performed in living and apoptotic evshRNA and casp6shRNA HeLa cells transiently transfected with CAD-GFP. The results are expressed as the mean +/− standard deviation (n = 20).

Besides caspase-3, in mammals there are two other effector caspases, caspase-6 and 7. Both proteins have some substrates in common with caspase-3, and both are involved in the regulation of nuclear changes during apoptosis, including nuclear lamina breakdown [9], [23]. To investigate the role of the different caspases in the immobilization of HMGB1 and CAD, we first determined the level of activation of effector caspases in MCF-7/pv and MCF-7/c3 treated with TNF-α and CHX. Procaspase-7 is processed both in apoptotic MCF-7/pv and MCF-7/c3 cells (Figure 7A). Therefore, caspase-7 cannot be responsible for the immobilization of HMGB1 and CAD in the nucleus of apoptotic cells. Indeed, procaspase-7 appeared to be processed to a greater extent in cells expressing caspase-3, suggesting that its cleavage may in part depend on caspase-3 activation, probably indirectly.

On the contrary, procaspase-6 is cleaved only in MCF-7/c3 apoptotic cells (Figure 7A). We then tested HMGB1 and CAD immobilization in HeLa cells depleted of caspase-6 by shRNA (Figure 7B). FRAP experiments performed in proliferating and apoptotic HeLa evshRNA and Casp6shRNA cells demonstrate that caspase-6 depletion does not affect HMGB1 and CAD mobility, both in proliferating and apoptotic cells (Figure 7C–D).

We conclude that both HMGB1 and CAD immobilization are specifically dependent on activation of caspase-3, but not of caspase-6 and 7.

## Discussion

We have investigated here, using epistasis analysis, five nuclear events that take place in mammalian cells during apoptosis (DNA laddering, histone H2B and H2AX phosphorylation, and the immobilization of CAD and HMGB1 proteins) in order to delineate whether these events constitute an ordered pathway in apoptosis, and thus whether any controls any other.

**Figure 8:**
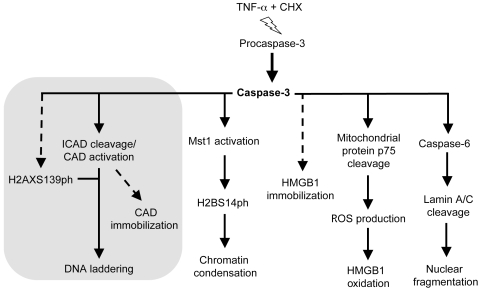
Cause-effect relationships in nuclear events during apoptosis. Broken lines indicate incomplete knowledge: intermediate steps might be missing, or causation is only inferred. Continuous lines indicate direct cause-effect relationships. The grey area groups events that appear interconnected downstream of caspase-3 actiavtion: histone H2AX phosphorylation, ICAD cleavage, CAD activation, DNA laddering and CAD immbilization.

Caspase-3 activation causes DNA laddering (via ICAD cleavage and CAD activation), the immobilization of CAD, and histone H2B phosphorylation (via Mst1 kinase activation) (Figure 8). Lu et al. [21] reported that, upon UV irradiation, JNK1 phosphorylates H2AX and activates caspase-3; the JNK inhibitor SP600125 or dominant negative JNK1 not only suppressed activation of JNK1 but also blocked H2AX phosphorylation and caspase-3 activation. However, the inhibition of caspase-3 did not reduce H2AX phosphorylation, leading to the suggestion that caspase-3 activation is independent from H2AX phosphorylation. In contrast to these published results, we find that in MCF-7 cells undergoing TNF-α induced apoptosis the phosphorylation of H2AX occurs only when caspase-3 is expressed and activated (Figures 4A and 8). We cannot exclude, however, that this difference is due to the specific mode of apoptosis induction (UV irradiation vs TNF-α plus cycloheximide) or the particular cell line used.

We find that also HMGB1 immobilization depends on caspase-3 activation (Figure 8). Our evidence rests on two observations: (1) the caspase-3 inhibitor z-DEVD-fmk abrogates HMGB1 immobilization in cells treated with TNF-α and CHX (data not shown), and (2) MCF-7 cells, which do not express caspase-3, do not immobilize HMGB1 upon apoptosis, unless the expression of caspase-3 is restored (Figure 3B).

Nuclear lamina breakdown depends on capase 6 activation [9], which in turns depends on caspase-3 activation (Figure 7). Thus, all five nuclear events depend on caspase-3 activation (Figure 8). We then examined the downstream cause-effect relationships.

It was previously known that CAD activation causes DNA laddering, and the absence of H2AX abrogates laddering [21] (Figure 8). Here we showed that the absence of H2B phosphorylation (via Mst1 knockdown) or the knockdown of Hmgb1 does not affect laddering. Conversely, laddering does not cause H2B phosphorylation (which occurs also in *Cad−/−* cells, [12], nor HMGB1 immobilization (HMGB1 is immobilized also in ICAD overexpressing cells, where no laddering is evident [4].

Elimination of H2B phosphorylation (via knockdown of Mst1) does not abrogate H2AX phosphorylation, CAD immobilization or HMGB1 immobilization (Figure 5). H2AX phosphorylation does not cause HMGB1 immobilization, at least in the presence of TSA (Figure 4). Knockdown of HMGB1 does not affect H2B phosphorylation, H2AX phosphorylation, or CAD immobilization (Figure 6).

In conclusion, this set of results imply that CAD activation, DNA laddering, and H2AX phosphorylation and probably CAD immobilization cluster together in a pathway (grey area in Figure 8). However, DNA laddering, nuclear lamina breakdown, H2B phosphorylation, and HMGB1 immobilization are independent endpoints. This suggests that these events and the “DNA laddering cluster” evolved independently, and thus each was subject to evolutionary pressure in its own right. Thus, the various endpoints might serve different purposes.

We previously argued that HMGB1 immobilization evolved to allow differential responses to programmed and accidental cell death [8]. In fact, apoptotic cells also oxidize HMGB1, via the production of reactive oxygen species (ROS) by mitochondria where protein p75 has been cleaved by caspase-3, and electron transport has been stalled [18]. We show here that HMGB1 immobilization does not depend on its oxidation (Figure 2); thus, even HMGB1 immobilization and oxidation appear to be separately controlled, albeit both downstream of caspase-3 activation (Figure 8).

Recently, we have also shown that HMGB1-nucleosome complexes released from late apoptotic cells are inflammatory and lead to the production of autoantibodies against histones and DNA, and thus the pathogenesis of systemic lupus erythematosus (SLE) [24]. Nucleosomes can only be released from apoptotic cells if the DNA has been cut; thus, DNA laddering may be responsible for anti-histone and anti-DNA autoantibody formation in autoimmune patients. Interestingly, *Cad−/−* mice have no overt phenotype; mice lacking DNAse II, the enzyme that degrades the chromatin of engulfed cells in macrophages, die because of constitutive production of interferon β [25], and *Dnase2−/− Ifnar1−/−* double mutant mice (that survive excessive interferon β production because they lack the receptor) develop chronic polyarthritis [26]. Finally, *Cad−/− Dnase2−/−* double mutant mice survive, but have impaired thymic development [2]. Although these pieces of information are sparse, the generation of nucleosome-sized chromatin pieces, and the inability to degrade them further, appear to cause inflammation and autoimmunity. Against this backdrop, there must be some countervailing advantage to DNA laddering, otherwise it would be lost in evolution. The link between HMGB1, DNA laddering and inflammation/immunity is of theoretical and practical interest, and deserves further investigation.

## Materials and Methods

### Cell culture and reagents

HeLa (ATCC), MCF-7/pv and MCF-7/c3 cells (courtesy of Dr. AD Thor, Department of Pathology, University of Colorado Health Sciences Center, Denver, CO) were cultured in Dulbecco's modified Eagle's medium (DMEM) supplemented with 10% foetal bovine serum (FBS from GIBCO), 100 IU/ml of penicillin and 100 µg/ml streptomycin, in 5% CO_2_ humidified atmosphere. Apoptosis was induced by treating the cells with 2 ng/ml human TNF-α (R&D) and 35 µM cycloheximide (Sigma). Where indicated, 100 µM z-DEVD-fmk (Sigma) was added to cells 1 hour before apoptosis induction, or 100 ng/ml TSA (Sigma) just prior apoptosis induction. Antibodies agaist H2BS14ph (Upstate) H2B (Upstate), anti-H3ac (Upstate), H3 (Abcam), H2AXS139ph (Upstate), HMGB1 (BD Pharmingen), Mst1 (Upstate), Caspase-3 (Chemicon), Caspase-6 (Santa Cruz Biotechnology), Caspase-7 (MBL International Corporation) were used in Western blotting.

### Plasmids

We generated the constructs containing BoxA, BoxB, BoxA+BoxB, BoxB+C-term fused in frame to the GFP by cloning into the XhoI/SacII sites of the vector pEGFP-N1 four different cassettes, produced by annealing the following pairs of primers using pEGFP-N1-HMGB1 as template:

- BoxA-GFP: BoxAfor 5′-GATCTCGAGATGGGCAAAGGAGATCCTAAG-3′ and BoxArev 5′-GGCCCGCGGCCCTTTGGGGGGATGTA-3′
- BoxB-GFP: BoxBfor 5′-GATCTCGAGATGGACCCCAATGCCCCC-3′ and BoxBre: 5′-GGCCCGCGGCACCCCCTTTTTCGCTGC-3′
- BoxA+B-GFP: BoxAfor and BoxBrev
- BoxB+C-term: BoxBfor and CTermrev 5′-GGCCCGCGGTTCATCATCATCATCTTCTTCTTCACT-3′

The annealed PCR products were digested with XhoI/SacII, purified on a 1.2% agarose gel and cloned into the pGFP-N1 vector. All constructs were checked by double strand sequencing.

A vector for HMGB1 silencing (pHMGB1shRNA) was generated by cloning annealed oligonucleotides derived from human HMGB1 cDNA (sense, 5′-GGATATTGCTGCATATCGA-3′) into pSuperior.puro empty vector (Invitrogen).

We also used the following plasmids:

- pEGFP-N1-HMGB1 [*4*]
- pEGFP-N1-mutHMGB1 [*16*]
- pEGFP-N1-CAD (courtesy of Dr. Gergely L. Lucaks) [5]
- pSuperMst1 (courtesy of Dr. Dae-Sik Lim)
- pRS vector (OriGene Technologies Inc., TR20003) and casp6shRNA pRS vector (TI329635)
- pEGFP-N1-HMGB1C22S, pEGFP-N1-HMGB1C44S, pEGFP-N1-HMGB1C105S, pEGFP-N1-HMGB1C22/44/105S [27]

### Transient and stable transfections

For transient trasfections, cells were transfected the day after seeding using FuGene 6 Trnsfection Reagent (Roche) with a ratio of 3 µl FuGene to 1 µg DNA, following the manufacturer's instructions.

For stable transfections, 300 000 HeLa cells were seeded in 10 cm dishes and co-transfected using FuGene with 6 µg of the plasmid of interest and 0.5 µg pEGFP-N1 to check for transfection efficiency. Fortyeight hours after transfection, 1.5 µg/ml of puromycin (Clontech) was added to the medium. After ten days of selection, single resistant clones were picked with sterile tips, amplified and analysed for protein expression by Western blot.

### Fluorescence recovery after photobleaching (FRAP)

To perform FRAP experiments, cells were grown in glass-bottom petri dishes (LabTek, Nunc), transiently transfected, and induced into apoptosis when indicated. Photobleaching experiments were performed on a Leica TCS SP2 AOBS confocal microscope equipped with a 63×/1.4 N.A. oil immersion objective at 37°C. To define the prebleaching plateau, five single-section 12-bit images were acquired with 6× zoom on a small area (47×12 µm, pixel size 0.093 µm) to maximize acquisition speed (107 ms/frame). Bleaching was performed with four 107 ms pulses using the 488 nm and 514 nm lines of an Ar laser (100 mW nominal output) at 90% power on an area of 1 µm radius. Fluorescence recovery was monitored collecting 100 single-section images at 107 ms intervals with low laser intensity (2% of the bleach intensity with the single 488 nm laser line, detection 520–650 nm). For quantitative analysis of fluorescence recovery, data were doubly normalized as described [28].

The mobile fraction of the protein was calculated by comparing the fluorescence in the bleached region after full recovery (F_final_) with that before bleaching (F_initial_) and just after bleaching (F_bleached_) (Figure 1B). In particular, the mobile fraction *R* is defined as:
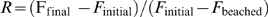



### Detection of DNA fragmentation

To analyse genomic DNA by gel electrophoresis, ∼500,000 proliferating and apoptotic cells were recovered, washed with PBS, resuspended in 350 µl Lysis buffer (100 mM Tris pH 8.5, 5 mM EDTA, 0.2% SDS, 200 mM NaCl) containing 500 µg/ml proteinase K and incubated for 4 hours at 56°C and 0,25 mg/ml RNase. DNA was precipitated with 2.5 vol of 100% EtOH and 300 mM Na-acetate pH 5.3, washed with 70% EtOH, resuspended in 30 µl of water, and analysed by electrophoresis on a 1.5% agarose gel.

### Statistical analysis

Pairwise comparisons between continuous data were done using unpaired two-tailed Student's t-test.

## Supporting Information

Figure S1 The knock-down of Mst1 has no effects on DNA laddering or HMGB1 and CAD mobility. (A) The knockdown of Mst1 (lanes 2: Mst1shRNA cells) or HMGB1 (lanes 3: HMGB1shRNA cells) does not affect nuclear DNA breakdown during apoptosis. (B) FRAP experiments were performed in living and apoptotic evshRNA and Mst1shRNA cells transfected with HMGB1-GFP. The results are expressed as the mean +/− standard deviation (n = 18). (C) FRAP experiments were performed in living and apoptotic evshRNA and Mst1shRNA cells transfected with CAD-GFP. The results are expressed as the mean +/− standard deviation (n = 25).
(1.04 MB TIF)
