# Supplementary figures and images for: Several Nuclear Events during Apoptosis Depend on Caspase-3 Activation but Do Not Constitute a Common Pathway
Source: PLoS One. 2009 Jul 29;4(7):e6234. doi: 10.1371/journal.pone.0006234 (PMC2713420; doi:10.1371/journal.pone.0006234)

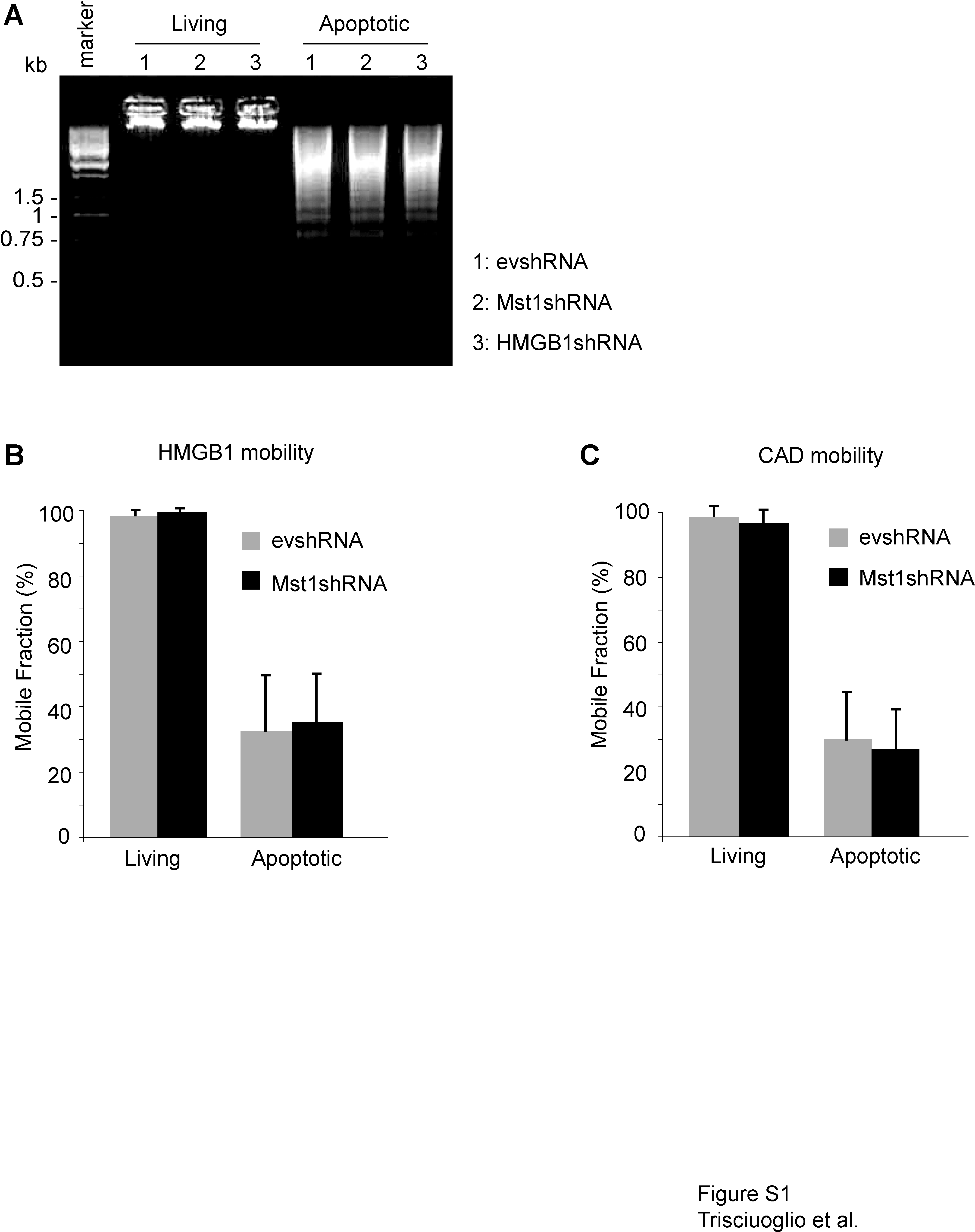

Supplement: Figure S1 — The knock-down of Mst1 has no effects on DNA laddering or HMGB1 and CAD mobility. (A) The knockdown of Mst1 (lanes 2: Mst1shRNA cells) or HMGB1 (lanes 3: HMGB1shRNA cells) does not affect nuclear DNA breakdown during apoptosis. (B) FRAP experiments were performed in living and apoptotic evshRNA and Mst1shRNA cells transfected with HMGB1-GFP. The results are expressed as the mean +/− standard deviation (n = 18). (C) FRAP experiments were performed in living and apoptotic evshRNA and Mst1shRNA cells transfected with CAD-GFP. The results are expressed as the mean +/− standard deviation (n = 25). (1.04 MB TIF) [file pone.0006234.s001.tif]
